# The effect of Inc parameter on VMAT radiotherapy plans quality for rectal cancer using Monaco TPS

**DOI:** 10.1002/acm2.14409

**Published:** 2024-06-24

**Authors:** Peng Zhou, Jia Luo, Xiaona Su, Chuan Chen

**Affiliations:** ^1^ Department of Oncology Daping Hospital, Army Military Medical University Chongqing China

**Keywords:** increment, Monaco treatment planning system, rectal cancer, volumetric modulated arc therapy

## Abstract

**Background:**

To investigate the effect of the Increment of gantry angle (Inc) parameter setting of the Monaco Treatment planning system (Monaco TPS) on the dosimetry and quality parameters of the volumetric modulated arc therapy (VMAT) program for rectal cancer.

**Methods:**

A retrospective analysis was conducted on 50 patients with rectal cancer who underwent intensity modulated radiation therapy using the Monaco TPS system from 2020 to 2021. Under the same optimization function configuration and other parameter settings, the Inc parameters in the VMAT radiotherapy plan were set to 10°, 20°, 30°, and 40°. The dose‐volume histogram (DVH) was used to evaluate the dose distribution of the target area and the radiation dose of the organs at risk (OAR). The differences in the dosimetry of the planning target volume (PTV) and OAR, as well as the gamma pass rate (GPR) were compared.

**Results:**

In terms of target dose, D98, *D*_min_, HI, and conformity index (CI) of Inc10 group was significantly lower than those of Inc20, 30, and 40 groups (*P* < 0.05), and D2 of Inc10 group was significantly higher than that of Inc20 group (*P* = 0.009). We also found CI of Inc20 and 30 were significantly better than that of Inc40 (both *P* < 0.05). In terms of OAR dose, the study found that the *D*_mean_, *D*_min_, *V*_50%_, *V*_45%_, and *V*_40%_ for the bladder of the Inc10 group were lower than those of the other groups (all *P* < 0.05), the *D*_mean_ for femoral head of the Inc20 group was lower than that of the Inc30 group (*P* < 0.05), and Inc20 showed a better protective effect on the femoral head. The MUs tend to decrease as the Inc parameter setting is increased. The monitor unit (MU) in Inc10 group were significantly higher than those in Inc20, Inc30, and Inc40 groups, and the MU of Inc20 group was significantly higher than that of Inc40 group (both *P* < 0.05). We found that for the 3%/3 mm and 2%/2 mm standards, the GPRs of each plan were > 90%, which met clinical requirements.

**Conclusions:**

Different settings of Inc parameters have varying degrees of impact on target dose, OAR dose, and machine MU. It is important for doctors to choose different Inc parameters according to different clinical needs.

**Abbreviations**
3D‐CRT: 3‐dimensional conformal radiotherapy therapy
CTV: clinical target volume
DVH: dose‐volume histogram
GPR: gamma pass rate
IMRT: intensity‐modulated radiation therapy
Inc: increment of gantry angle
MLC: multi‐leave collimators
Monaco TPS: Monaco Treatment planning system
MU: monitor unit
OAR: organs at risk
PTV: planning target volume
VMAT: volumetric modulated arc therapy

## 1 BACKGROUND

Rectal cancer is one of the most common gastrointestinal cancers. It has become the third most common cancer in the world and its incidence rate is increasing.^1^, ^2^ The treatment options include surgery, radiation therapy, chemotherapy, and targeted therapy.^3^ Radiotherapy plays an important role in the treatment of rectal cancer, preoperative neoadjuvant radiotherapy, postoperative adjuvant radiotherapy, concurrent chemoradiotherapy for locally advanced rectal cancer, and radiotherapy for local‐regional recurrence after radical resection of rectal cancer all play important roles in the treatment of rectal cancer.^4^ To some extent, radiotherapy can reduce the rate of local tumor recurrence and improve the rate of local control.^5^ Therefore, radiotherapy is a better treatment method for preventing local recurrence because of its high efficacy and a short course of treatment, it can achieve better local control effect and is highly safe.^6^, ^7^

Because rectal cancer is so close to vital organs like the small intestine and bladder, traditional external beam radiation cannot deliver enough doses to the target area due to large position movement and organ restriction, resulting in a higher recurrence rate.

In most regions of China, intensity‐modulated radiation therapy (IMRT) is the preferred method for rectal cancer radiotherapy. It has good target conformity index when compared to traditional 3‐dimensional conformal radiotherapy therapy (3D‐CRT).^8^ However, the target area of rectal cancer is large and requires more beams, so the IMRT treatment duration is longer, which may lead to patient position movement during treatment, affecting the accuracy of treatment. Volumetric modulated arc therapy (VMAT) is a type of IMRT that completes radiotherapy by rotating the gantry, moving the collimator and multi leaf collimators (MLC) during the beam delivery, and then combining this with a change in dose rate.^9^, ^10^ In the context of target coverage, VMAT technology has proven to be an effective way to reduce implementation duration.^8^ In comparison to IMRT and 3D CRT, VMAT significantly reduced the dose of organs at risk (OAR) in several clinical studies.^11^, ^12^, ^13^ In addition, compared to IMRT, VMAT can improve the quality of radiotherapy plans, reduce radiotherapy related adverse reactions, and have a therapeutic effect not inferior to IMRT in rectal cancer radiotherapy, it also has the advantages of uniform dosage and short treatment time, and the application of VMAT in rectal cancer radiotherapy is gradually increasing, with good prospects.^2^, ^12^, ^13^, ^14^, ^15^, ^16^

The Increment (Inc) parameter setting is a mechanical limiting parameter that is unique to developing VMAT radiotherapy plans with the Monaco treatment planning system (TPS). In VMAT radiotherapy plans, it represents the angle at which the gantry is turned during a leaf movement of MLC. Inc parameter controls the number of generated static gantry positions or sectors in VMAT radiotherapy plan. A planning study found that plans with larger Inc parameters outperform plans with smaller Inc parameters in dose coverage, uniformity index, consistency index, and normal tissue sparing.^17^ There are no studies on the impact of Inc parameter settings on the quality of VMAT radiotherapy plans for rectal cancer now, except for esophagus cancer and cervix.^17^, ^18^ The primary goal of this study is to determine the effect of Inc parameter settings on the quality of VMAT radiotherapy plans to better guide the clinical application of rectal cancer radiotherapy.

## 2 METHODS

### 2.1 Study design and participants

In this retrospective study, 50 rectal cancer patients who underwent IMRT after rectal cancer surgery at the People’s Liberation Army of China (PLA) Army Medical Center from January to October 2021 were randomly selected, including 34 males and 16 females, with an average age of 59 years. All patients were diagnosed as rectal adenocarcinoma by colonoscopy biopsy and histopathology, with stage II–III and no distant organ metastasis. This study was approved by the Ethics Committee. All patients have signed informed consent forms.

### 2.2 Posture fixation and CT scanning

The patients were immobilized in a supine position with Belly‐Pelvis Mask. A 16‐ slice spiral Big Bore CT (Philips, Netherlands) with a slice thickness of 5 mm was used to scan the image. The scanning range was from the diaphragm's lower horizontal boundary to the superior of the femur's third segment, and the images were sent to the Monaco TPS (Elekta Sweden, version 5.11).

### 2.3 Treatment plan generation

**FIGURE 1:**
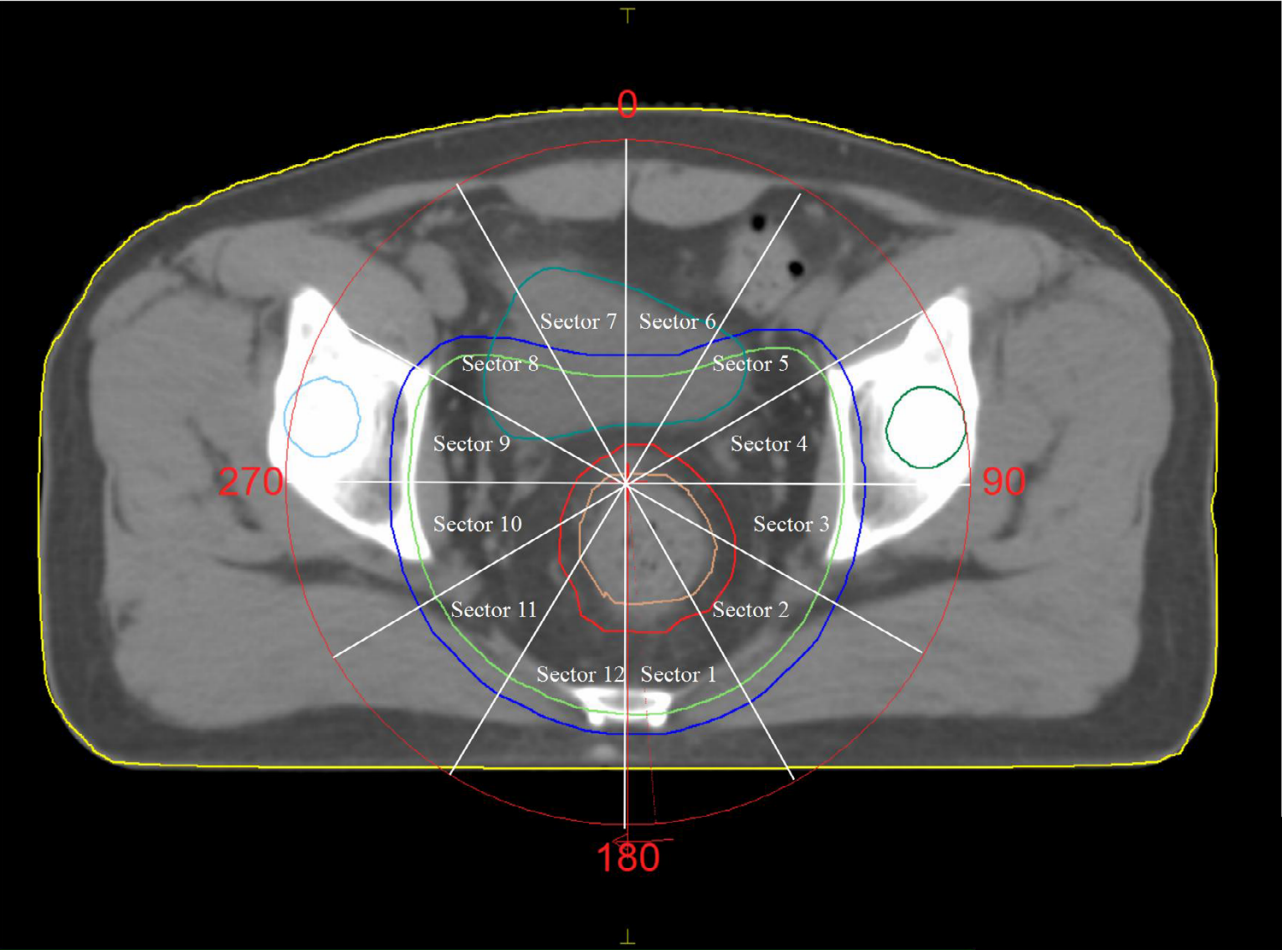
Sectors for 360° arc length.

The clinical target volume (CTV) and OAR were delineated and confirmed by an experienced oncology radiotherapy physician. Seven millimeters to 1 cm margin was added laterally and 1 cm margin was added superiorly and inferiorly to the CTV to create planning target volume (PTV). OAR organs included the intestines, bladder, and femoral head. The plan was designed on the Monaco TPS, with 8 MV x‐ray VMAT radiotherapy for all programs per patient at a dose of 50 Gy/25Fraction. The PTV center was selected as the isocenter. The coplanar arc design was employed, and reverse optimized with the same function configuration and parameters (Figure 1). Inc parameters were set to 10°, 20°, 30°, and 40°, in that order. Inc10, Inc20, Inc30, and Inc40 are the names given to four different VMAT plans.

### 2.4 Plan evaluation

The plan acceptance criterion is that 95% of the PTV corresponds to 100% of the prescribed dose. The dose‐volume histogram (DVH) was used to compare the dosimetry parameters of the target and OAR, as well as the conformity and uniformity of the dose distribution between the four VMAT plans. The comparison parameters included the following: target volume *D*_98%_, *D*_2%_, *D*_50%_, *D*_max_, *D*_mean_, *D*_min_, *V*_107%_, homogeneity index (HI),^19^ left and right femoral head of OAR *V*_45%_, *V*_40%_, *D*_max_, *D*_mean_, *D*_min_, bladder *V*_50%_, *V*_45%_, *V*_40%_, *D*_max_, *D*_mean_, *D*_min_, MUs.

CI = (*V*_tref_/*V*_t_) × (*V*_tref_/*V*_ref_), where *V*_tref_ represents the volume of the target covered by the reference (prescribed dose) isodose, *V*_t_ represents the target volume, and *V*_ref_ represents the volume of the reference isodose. The higher the CI value, the better the conformity.

HI = *D*_5_/*D*_95_, where *D*_5_ was the dose that covered 5% of the target volume and *D*_95_ was the dose that covered 95% of the target volume. The lower the HI value, the more uniform the dose.^20^, ^21^

### 2.5 Dose verification

The MOSAIQ system was used to schedule and verify all plans on ELekta AXESSE model accelerators. The ArcCHECK‑3DVH system (Sun Nuclear Corporation [SNC], Melbourne, Florida, USA) was used for dosimetric verification of all treatment plans. In Monaco TPS, transplant all plans into the Arccheck phantom to recalculate the dose, record it as the VMAT plan's quality assurance plan, and export the radiotherapy (RT) plan and RT Dose files. The Arccheck was installed in accordance with the user manual. Following background correction, actual measurements for each VMAT plan were taken, and the actual measured dose was obtained. In the SNC patient software, the difference between the calculated dose and the actual measured dose was compared, and the gamma pass rate (GPR) was recorded at a threshold of 10% under different global gamma analysis conditions (3 mm/3%, 2 mm/2%, 1 mm/1%).^22^

### 2.6 Statistical analysis

All dosimetrical parameters among four groups were represented with median and interquartile range. Friedman test was used to evaluate the difference in dosimetrical parameters among four groups. Nemenyi test was utilized to perform stepwise comparison between any two groups. Nemenyi test was performed with R package “PMCMRplus” (version 1.9.9) with correction for multiple comparisons by using Bonferroni method.

## 3 RESULTS

### 3.1 Target doses

There were no statistically significant differences in *D*_50_, *D*_max_, *D*_mean_, and *V*_107%_ of the target areas among the four groups (all *P* > 0.5). The D98 of Inc10 group was significantly lower than those of Inc20, 30, and 40 groups (*P* < 0.05), while there was no significant difference among the Inc20, 30, and 40 groups (*P* > 0.05); The D2 of Inc10 group was significantly higher than that of Inc20 group (*P* = 0.009), and there was no difference among the other groups (*P* > 0.05); The *D*_min_, of Inc10 group was significantly lower than those of Inc20, 30, and 40 groups (*P* < 0.05), while there were no significant differences among Inc20, 30, and 40 groups (*P* > 0.05). The HI of Inc10 group was significantly worse than that of Inc20, 30, and 40 groups (*P* < 0.05), while there were no significant differences among Inc20, 30, and 40 groups (*P* > 0.05); The CI of Inc10 group was significantly better than that of Inc20, 30, and 40 groups (*P* < 0.05), while the CI of Inc20 and 30 were significantly better than that of Inc40 (*P* < 0.05). There were no significant differences in CI among Inc20 and Inc30 groups (*P* = 1.00) (Table 1).

**TABLE 1:** Results of parameter statistical analysis of plans base on different Inc parameter design.

						adj‐*P* value					
	Dosimetric indicators	Inc10	Inc20	Inc30	Inc40	Inc10 versus 20	Inc10 versus 30	Inc10 versus 40	Inc20 versus 30	Inc20 versus 40	Inc30 versus 40
PTV	*D*_98%_/cGy	5046.850 (5013.250,5056.325)	5075.400 (5069.375‐5083.575)	5075.450 (5069.150,5085.375)	5079.450 (5071.725,5093.100)	<0.001	<0.001	<0.001	0.974	0.455	0.722
	*D*_2%_/cGy	5300.900 (5283.600,5314.625)	5287.650 (5272.300‐5300.125)	5288.000 (5274.775,5299.375)	5292.900 (5282.225,5301.900)	0.009	0.144	0.321	0.745	0.479	0.974
	*D*_50%_/cGy	5170.400 (5155.200,5174.125)	5164.150 (5159.550,5172.675)	5165.200 (5158.800,5172.550)	5168.450 (5161.725,5176.025)	0.993	0.967	0.264	0.883	0.156	0.527
	*D*_min_/cGy	4694.100 (4580.500,4772.625)	4806.400 (4694.225,4895.300)	4812.100 (4723.250,4902.850)	4852.250 (4710.675,4898.450)	<0.001	<0.001	<0.001	0.745	0.866	0.996
	*D*_max_/cGy	5477.450 (5463.925,5492.700)	5467.450 (5449.325,5479.825)	5465.000 (5450.150,5479.775)	5467.900 (5457.000,5484.050)	0.132	0.745	0.949	0.651	0.364	0.967
	*D*_mean_/cGy	5173.000 (5154.950,5175.375)	5166.400 (5161.325,5177.050)	5168.500 (5161.200,5176.750)	5170.600 (5164.975,5178.850)	0.926	0.745	0.282	0.364	0.077	0.866
	*V*_107%_	0.340 (0.140,0.580)	0.230 (0.110,0.355)	0.250 (0.130,0.328)	0.240 (0.172,0.405)	0.084	0.699	0.527	0.577	0.745	0.993
	HI	1.040 (1.040,1.040)	1.030 (1.030,1.030)	1.030 (1.030,1.030)	1.030 (1.030,1.038)	<0.001	<0.001	<0.001	0.999	0.967	0.926
	CI	0.850 (0.840,0.860)	0.810 (0.790,0.820)	0.810 (0.810,0.820)	0.810 (0.780,0.810)	<0.001	<0.001	<0.001	1.000	0.005	0.004
	Mu	840.480 (786.805,887.527)	743.605 (708.092,790.222)	736.505 (691.920,761.137)	706.725 (669.413,758.058)	<0.001	<0.001	<0.001	0.052	0.002	0.699
Right femoral head	V45/%	0.010 (0.000,0.048)	0.010 (0.000,0.040)	0.015 (0.000,0.050)	0.005 (0.000,0.028)	0.985	0.913	0.183	0.745	0.342	0.038
	V40/%	0.410 (0.165,0.652)	0.310 (0.135,0.672)	0.400 (0.110,0.637)	0.280 (0.110,0.550)	0.213	0.866	0.005	0.651	0.503	0.052
	*D*_max_/cGy	4611.550 (4470.175,4731.450)	4613.700 (4446.650,4738.150)	4617.200 (4381.950,4805.100)	4583.950 (4384.400,4732.675)	1.000	0.789	0.898	0.829	0.866	0.364
	*D*_mean_/cGy	1637.000 (1519.425,1803.500)	1631.400 (1533.225,1702.400)	1709.900 (1624.375,1814.300)	1925.950 (1826.525,1996.275)	0.651	0.213	<0.001	0.010	<0.001	<0.001
	*D*_min_/cGy	445.350 (371.950,587.625)	469.000 (340.150,593.450)	357.850 (312.050,493.325)	434.650 (319.050,603.050)	1.000	<0.001	0.990	0.001	0.980	<0.001
Left femoral head	V45/%	0.010 (0.000,0.050)	0.000 (0.000,0.068)	0.005 (0.000,0.100)	0.000 (0.000,0.050)	0.866	0.789	0.926	0.321	0.999	0.408
	V40/%	0.440 (0.133,0.660)	0.335 (0.082,0.730)	0.380 (0.133,0.810)	0.275 (0.060,0.648)	0.183	0.479	0.057	0.003	0.958	<0.001
	*D*_max_/cGy	4571.100 (4407.025,4692.175)	4582.750 (4324.675,4753.525)	4590.750 (4399.300,4811.775)	4540.650 (4295.850,4809.975)	1.000	0.077	0.967	0.063	0.980	0.022
	*D*_mean_/cGy	1708.000 (1487.800,1884.500)	1604.300 (1474.200,1713.250)	1721.900 (1659.325,1802.350)	1902.500 (1812.450,1988.650)	0.042	1.000	<0.001	0.034	<0.001	<0.001
	*D*_min_/cGy	411.100 (295.000,558.800)	427.650 (313.225,544.000)	369.500 (273.325,498.325)	388.200, (291.875,556.000)	0.980	0.077	0.866	0.027	0.980	0.008
Bladder	V50/%	30.340 (24.303,34.445)	32.915 (26.310,38.280)	33.050 (26.205,37.955)	33.610 (26.440,38.330)	<0.001	<0.001	<0.001	0.132	0.019	0.883
	V45/%	41.695 (33.742,45.597)	44.050 (36.538,47.820)	45.015 (37.513,49.225)	45.895 (37.320,49.733)	<0.001	<0.001	<0.001	0.093	0.002	0.552
	V40/%	48.800 (40.782,52.795)	51.585 (44.932,55.448)	53.365 (47.680,56.957)	54.810 (48.725,58.940)	0.001	<0.001	<0.001	0.034	<0.001	0.156
	*D*_max_/cGy	5405.200 (5375.275,5428.325)	5388.250 (5358.575,5404.100)	5384.650 (5357.750,5416.000)	5387.500 (5361.725,5402.250)	0.004	0.077	0.022	0.745	0.949	0.967
	*D*_mean_/cGy	3535.350 (3363.650,3861.500)	3865.750 (3580.000,4015.950)	3866.450 (3584.725,4044.450)	3915.100 (3628.300,4116.275)	<0.001	<0.001	<0.001	0.156	0.004	0.552
	*D*_min_/cGy	1212.850 (1045.375,1354.900)	1577.950 (1399.900,1777.350)	1608.950 (1444.975,1838.925)	1432.200 (1300.850,1570.900)	<0.001	<0.001	<0.001	0.183	0.156	<0.001

Abbreviations: CI, conformity index; HI, homogeneity index; INC, increment of gantry angle; MU, monitor unit; PTV, planning target volume.

### 3.2 OAR dose

For the right femoral head, there was no statistically significant difference in *D*_max_ among the four groups (*P* > 0.05); the V45 of Inc40 group was lower than that of Inc30 group (*P* = 0.038); the V40 of Inc40 group was lower than that of Inc10 group (*P* = 0.005); the *D*_mean_ of Inc10, 20, and 30 groups were lower than that of Inc40 group (*P* < 0.05), and the *D*_mean_ of Inc20 group was lower than that of Inc30 group (*P* < 0.05); the *D*_min_ of Inc30 group was lower than that of Inc10, 20, and 40 groups (*P* < 0.05). For the left femoral head, there were no statistically significant difference in V45 among the four groups (*P* > 0.05); the V40 of Inc20 and 40 groups were lower than that of In30 group (*P* < 0.05); The *D*_max_ of Inc40 group was lower than that of Inc30 group; the *D*_mean_ of Inc10, 20, and 30 groups was lower than that of Inc40 group, and the *D*_mean_ of Inc20 group was lower than that of Inc10 and 30 groups (*P* < 0.05). There was no significant difference in *D*_mean_ between Inc10 and 30 groups (*P* > 0.05); The *D*_min_ of Inc30 group was lower than that of In20 and 40 groups (*P* < 0.05). For the bladder, the V50, V45, and V40 of Inc10 group were lower than those of Inc20, 30, and 40 groups (*P* < 0.05), the V50, V45, and V40 of Inc20 group were lower than those of Inc40 group (*P* < 0.05), and the V40 of Inc20 group was lower than that of Inc30 group; The *D*_max_ of Inc20 and 40 groups was lower than that of Inc10 group (*P* < 0.05); The *D*_mean_ and *D*_min_ of Inc10 group were lower than those of Inc20, 30, and 40 groups (*P* < 0.05). The *D*_mean_ of Inc20 group was lower than that of Inc40 group (*P* < 0.05), and the *D*_min_ of Inc40 group was lower than that of Inc30 group (*P* < 0.05) (Table 1).

### 3.3 MUs

The MU in Inc10 group were significantly higher than those in Inc20, Inc30, and Inc40 groups (*P* < 0.05). The MU of Inc20 group was significantly higher than that of Inc40 group (*P* < 0.05), while there was no significant difference among the other groups (*P* > 0.05) (Table 1).

### 3.4 GPR

The results showed that for the 3%/3 mm and 2%/2 mm standards, the GPRs of each plan were > 90%, which met clinical requirements. For the 3%/3 mm standard, the GPRs of Inc20 and Inc30 group were higher than those of Inc10 and Inc40 groups (*P* < 0.05), and there was no significant difference between Inc20 and Inc30 group, and between Inc10 and Inc40 group (*P* > 0.05); For the 2%/2 mm and 1%/1 mm standards, the GPR of Inc20, Inc30, and Inc40 were higher than that of Inc10 (*P* < 0.05), while the GPR of Inc30 was higher than those of Inc20 and Inc40 (*P* < 0.05) (Table 2).

**TABLE 2:** Gamma passing rates of plans base on different Inc parameter design.

					adj‐*P* value					
	Inc10	Inc20	Inc30	Inc40	Inc10 versus 20	Inc10 versus 30	Inc10 versus 40	Inc20 versus 30	Inc20 versus 40	Inc30 versus 40
3%/3 mm	99.000 (98.700,99.400)	99.600 (99.400,99.700)	99.800 (99.625,99.900)	99.200 (98.900,99.575)	<0.001	<0.001	0.789	0.063	<0.001	<0.001
2%/2 mm	93.500 (92.050,94.675)	95.200 (94.325,96.175)	96.950 (95.925,97.650)	95.400 (94.175,96.250)	<0.001	<0.001	0.001	<0.001	0.883	<0.001
1%/1 mm	66.050 (63.750,68.150)	70.450 (68.275,72.575)	73.700 (71.900,76.350)	71.300 (69.825,73.350)	<0.001	<0.001	<0.001	<0.001	0.626	0.001

Abbreviation: Inc, increment of gantry angle.

## 4 DISCUSSION

VMAT is a linear accelerator that modifies the MLC shape and dose rate as the gantry moves in order to improve target conformity and dose uniformity. Inc is a parameter setting that is unique to the Monaco TPS VMAT plan. It represents the angle generated by the gantry after back‐and‐forth movement of MLC in VMAT, with a setting range of 3°−60°.^23^ In general, too small Inc parameter increased wear of MLC, while too large Inc parameter reduces plan quality. Reasonable Inc parameter can help a VMAT plan for rectal cancer achieve better dose distribution. There are currently no relevant reports on the impact of Inc parameter settings in Monaco TPS on the quality of VMAT plans for rectal cancer. We investigated the effect of the Inc parameter on the dosimetry of VMAT treatment in rectal cancer, as well as the planned validation outcomes in this study. When the Inc parameters were set to 20° and 30°, there was no significant difference in the *D*_max_, *D*_min_, and *D*_mean_ of the target volume between the two groups. Furthermore, both groups had good target conformity, dose uniformity, and machine MUs. Different settings of Inc parameters have varying degrees of impact on target dose, OAR dose, and machine MUs. It is recommended that clinical doctors choose appropriate Inc parameters based on different clinical needs and use Monaco TPS to develop a rectal cancer VMAT plan.

In this study, first, we found that Inc10 has the advantage of lower target D98 and higher *D*_min_, while there is no significant difference among Inc20, Inc30, and Inc40. This means that smaller Inc has better control over the maximum and minimum doses in the target area, avoiding excessive dose hotspots. This may be related to the influence of Inc parameter settings on the beam output of the linear accelerator, frequent back‐and‐forth movement of MLC, and thus affecting the dose rate of the linear accelerator. Second, the conformity of the target decreased with the increase of Inc parameter. Inc10 has the best conformity of target, while Inc20 and Inc30 have no statistically significant difference in conformability of target, but were better than Inc40. This indicates that the smaller the Inc parameter settings, the better the conformity of target. Monaco divides the plan angle into equal sectors based on the selected increment. In each sector, MLC moves unidirectionally. Therefore, the lower the increment selected, the more sectors there are, resulting in more MLC sweeps.^18^ This corresponds to the target's shape change. A smaller Inc parameter can be recommended for plans that require good conformity. Third, we also found the target dose uniformity of Inc10 was lower than that of Inc20, Inc30, and Inc40. It is possible that the MLC frequency transformation affects the linac output and dose rate, resulting in poor dose uniformity in the target area. The doctor has the option to choose conformity and dose uniformity based on the individual patient's situation and the purpose of treatment.

Further analysis revealed that different settings of Inc parameters resulted in different changes in OAR dose. The study found that the *V*_50%_, *V*_45%_, *V*_40%_, *D*_mean_ and *D*_min_ of the bladder in the Inc10 group were slightly lower than those in the other groups, the *D*_mean_ of the femoral head in the Inc20 group was lower than that in the Inc30 group, and Inc20 showed a better protective effect on the femoral head. The potential cause is the smaller the Inc parameter, the better the target conformity. Although we recommend choosing Inc20 in our study, we should still choose the appropriate Inc parameter based on the actual situation of each plan. This reduced the dose of OAR exposure near the target area. Although there were differences in OAR doses among the four plans, the differences were minor. Given the higher doses tolerated of the bladder and femoral head, these differences have little effect on them.

The Inc parameter can also have an effect on the MUs. The MUs tend to decrease as the Inc parameter setting is increased. The higher the frequency of MLC scanning, the smaller the Inc parameter setting. The greater the MUs, the longer the duration of MLC scanning. MUs were found to be positively correlated with treatment duration in studies. Excessive MUs may also increase whole‐body low‐dose irradiation.^24^, ^25^ When the Inc parameters were set to 20°, 30°, and 40°, the treatment durations were less than that of Inc10°.

Plan validation results revealed that stricter judging criteria reduced the GPR for all plans significantly. The GPRs of the Inc20 and Inc30 were higher than those of the Inc10, and Inc40 for the 3%/3 mm. Inc30 had the highest GPR for the 1%/1 mm and 2%/2 mm standard. According to the TG218 study, IMRT should meet the gamma analysis standard of 3%/3 mm with a pass rate ≥ 95%, as well as the gamma analysis standard of 2%/2 mm with a pass rate ≥ 90%.^23^ The 1%/1 mm gamma analysis standard was used for data analysis under specific conditions but was not explicitly required by the standard.^26^ The dose validation results in this study indicated that all plans met the clinical use criteria.

This study has some limitations. First, this study is a single center, retrospective study, and the results require prospective, multicenter trials to validate. Second, the subjects of this study are Chinese patients. Future research needs to increase the number and ethnicity of patients.

## 5 CONCLUSION

In summary, different Inc parameter affect the target dose, OAR dose, and MUs in different ways. When developing a VMAT plan for rectal cancer using Monaco TPS, it is important for doctors to choose different Inc parameters according to different clinical needs.

## AUTHOR CONTRIBUTIONS

Concept and design: All authors (Zhou Peng; Luo Jia; Su Xiaona; Chuan Chen). Acquisition, analysis, or interpretation of data: All authors. Drafting of the manuscript: All authors. Critical revision of the manuscript for important intellectual content: All authors. Statistical analysis: Luo Jia. All authors read and approved the final manuscript. Chuan Chen is the guarantor and had full access to all the data in the study, takes full responsibility for the data integrity and the data analysis's accuracy, and had final responsibility for the decision to submit for publication.

## CONFLICT OF INTEREST STATEMENT

The authors declare that they have no competing interests.

## ETHICS APPROVAL

All procedures performed in studies involving human participants were in accordance with the Ethics Committee of the Army Medical Center of PLA (No. 2022−87) and with the 1964 Helsinki declaration and its later amendments or comparable ethical standards. This study was approved by the Ethics Committee of the Army Medical Center of PLA (No. 2022−87). Written informed consent was obtained from all subjects and/or their legal guardian(s).

## CONSENT FOR PUBLICATION

Not applicable.

## Data Availability

All data generated or analyzed during this study are included in this published article.

## References

[1] Keller DS , Berho M , Perez RO , Wexner SD , Chand M . The multidisciplinary management of rectal cancer. Nat Rev Gastroenterol Hepatol. 2020;17(7):414‐429. 32203400 10.1038/s41575-020-0275-y

[2] Hanna CR , Slevin F , Appelt A , et al. Intensity‐modulated radiotherapy for rectal cancer in the UK in 2020. Clin Oncol (R Coll Radiol). 2021;33(4):214‐223. 33423883 10.1016/j.clon.2020.12.011 PMC7985673

[3] Kane C , Glynne‐Jones R . Should we favour the use of 5×5 preoperative radiation in rectal cancer. Cancer Treat Rev. 2019;81:101908. 31683174 10.1016/j.ctrv.2019.101908

[4] Ha Thi HT , Duong HQ , Hong S . Emerging roles of noncoding RNAs in the response of rectal cancer to radiotherapy. Int J Oncol. 2021;58(3):344‐358. 33650664 10.3892/ijo.2021.5175

[5] Sauer R , Liersch T , Merkel S , et al. Preoperative versus postoperative chemoradiotherapy for locally advanced rectal cancer: results of the German CAO/ARO/AIO‐94 randomized phase III trial after a median follow‐up of 11 years. J Clin Oncol. 2012;30(16):1926‐1933. 22529255 10.1200/JCO.2011.40.1836

[6] Jin F , Luo H , Zhou J , et al. Dose‐time fractionation schedules of preoperative radiotherapy and timing to surgery for rectal cancer. Ther Adv Med Oncol. 2020;12:1758835920907537. 32165928 10.1177/1758835920907537 PMC7052459

[7] Fokas E , Appelt A , Glynne‐Jones R , et al. International consensus recommendations on key outcome measures for organ preservation after (chemo)radiotherapy in patients with rectal cancer. Nat Rev Clin Oncol. 2021;18(12):805‐816. 34349247 10.1038/s41571-021-00538-5

[8] Viani G , Hamamura AC , Faustino AC . Intensity modulated radiotherapy (IMRT) or conformational radiotherapy (3D‐CRT) with conventional fractionation for prostate cancer: is there any clinical difference? Int Braz J Urol. 2019;45(6):1105‐1112. 31808397 10.1590/S1677-5538.IBJU.2018.0842 PMC6909869

[9] Lyu Q , O'Connor D , Ruan D , Yu V , Nguyen D , Sheng K . VMAT optimization with dynamic collimator rotation. Med Phys. 2018;45(6):2399‐2410. 29659018 10.1002/mp.12915 PMC5997563

[10] Verbakel WF , Cuijpers JP , Hoffmans D , Bieker M , Slotman BJ , Senan S . Volumetric intensity‐modulated arc therapy vs. conventional IMRT in head‐and‐neck cancer: a comparative planning and dosimetric study. Int J Radiat Oncol Biol Phys. 2009;74(1):252‐259. 19362244 10.1016/j.ijrobp.2008.12.033

[11] Shaffer R , Morris WJ , Moiseenko V , et al. Volumetric modulated arc therapy and conventional intensity‐modulated radiotherapy for simultaneous maximal intraprostatic boost: a planning comparison study. Clin Oncol (R Coll Radiol). 2009;21(5):401‐407. 19268554 10.1016/j.clon.2009.01.014

[12] Engels B , Platteaux N , Van den Begin R , et al. Preoperative intensity‐modulated and image‐guided radiotherapy with a simultaneous integrated boost in locally advanced rectal cancer: report on late toxicity and outcome. Radiother Oncol. 2014;110(1):155‐159. 24239243 10.1016/j.radonc.2013.10.026

[13] Dröge LH , Weber HE , Guhlich M , et al. Reduced toxicity in the treatment of locally advanced rectal cancer: a comparison of volumetric modulated arc therapy and 3D conformal radiotherapy. BMC Cancer. 2015;15:750. 26486986 10.1186/s12885-015-1812-x PMC4617910

[14] Yang Y , Liu Q , Jia B , et al. Preoperative volumetric modulated arc therapy with simultaneous integrated boost for locally advanced distal rectal cancer. Technol Cancer Res Treat. 2019;18:1533033818824367. 30803368 10.1177/1533033818824367 PMC6373990

[15] Yamashita H , Ishihara S , Nozawa H , et al. Comparison of volumetric‐modulated arc therapy using simultaneous integrated boosts (SIB‐VMAT) of 45 Gy/55 Gy in 25 fractions with conventional radiotherapy in preoperative chemoradiation for rectal cancers: a propensity score case‐matched analysis. Radiat Oncol. 2017;12(1):156. 28934968 10.1186/s13014-017-0894-9 PMC5607844

[16] Song Y , Wang Q , Jiang X , Liu S , Zhang Y , Bai S . Fully automatic volumetric modulated arc therapy plan generation for rectal cancer. Radiother Oncol. 2016;119(3):531‐536. 27131593 10.1016/j.radonc.2016.04.010

[17] Nithya L , Raj NA , Rathinamuthu S , Sharma K , Pandey MB . Influence of increment of gantry angle and number of arcs on esophageal volumetric modulated arc therapy planning in Monaco planning system: a planning study. J Med Phys. 2014;39(4):231‐237. 25525311 10.4103/0971-6203.144488 PMC4258731

[18] Chen A , Li Z , Chen L , Lin M , Li B , Chen F . The influence of increment of gantry on VMAT plan quality for cervical cancer. J Radiation Res Appl Sci. 2019;12(1):447‐454.

[19] Bakkal BH , Elmas O . Dosimetric comparison of organs at risk in 5 different radiotherapy plans in patients with preoperatively irradiated rectal cancer. Medicine. 2021;100(1):e24266. 33429836 10.1097/MD.0000000000024266 PMC7793361

[20] Karpf D , Sakka M , Metzger M , Grabenbauer GG . Left breast irradiation with tangential intensity modulated radiotherapy (*t*‐IMRT) versus tangential volumetric modulated arc therapy (*t*‐VMAT): trade‐offs between secondary cancer induction risk and optimal target coverage. Radiat Oncol. 2019;14(1):156. 31477165 10.1186/s13014-019-1363-4 PMC6721379

[21] Liu P , Liu G , Wang G , et al. Comparison of dosimetric gains provided by intensity‐modulated radiotherapy, volume‐modulated arc therapy, and helical tomotherapy for high‐grade glioma. Biomed Res Int. 2020;2020:4258989. 32258121 10.1155/2020/4258989 PMC7109582

[22] Huang M , Huang D , Zhang J , Chen Y , Xu B , Chen L . Preliminary study of clinical application on IMRT three‐dimensional dose verification‐based EPID system. J Appl Clin Med Phys. 2017;18(4):97‐105. 10.1002/acm2.12098 PMC5875845 28594085

[23] Glitzner M , Crijns SP , de Senneville BD , Lagendijk JJ , Raaymakers BW . On the suitability of Elekta's agility 160 MLC for tracked radiation delivery: closed‐loop machine performance. Phys Med Biol. 2015;60(5):2005‐2017. 25675279 10.1088/0031-9155/60/5/2005

[24] Clark CH , Bidmead AM , Mubata CD , Harrington KJ , Nutting CM . Intensity‐modulated radiotherapy improves target coverage, spinal cord sparing and allows dose escalation in patients with locally advanced cancer of the larynx. Radiother Oncol. 2004;70(2):189‐198. 15028407 10.1016/j.radonc.2003.10.012

[25] Hall EJ , Wuu CS . Radiation‐induced second cancers: the impact of 3D‐CRT and IMRT. Int J Radiat Oncol Biol Phys. 2003;56(1):83‐88. 12694826 10.1016/s0360-3016(03)00073-7

[26] Miften M , Olch A , Mihailidis D , et al. Tolerance limits and methodologies for IMRT measurement‐based verification QA: recommendations of AAPM task group no. 218. Med Phys. 2018;45(4):e53‐e83. 29443390 10.1002/mp.12810

